# Artificial Lactation

**Published:** 1860-10

**Authors:** 


**Artificial Lactation.**

We are in receipt of an essay of six pages, on "Artifical Lactation," read before the "Indiana State Medical Society," May, 1860, By Dr. Charles M. Wetherill.

From the comparative analyses of human and cow's milk quoted in the essay, the usual preparation of cow's milk is a rational one. Water or some other diluent is necessary to lesson the proportion of casien and butter, and the addition of sugar and salt, to increase the proportion of these ingredients in cow's milk, in order to make it resemble in its constituents, the human milk. In the communication, it is advised to use milk sugar in the preparation of cow's milk for infants, instead of cane sugar.

